# Supplementary material for: Machine Learning Approach to Metabolomic Data Predicts Type 2 Diabetes Mellitus Incidence
Source: Int J Mol Sci. 2024 May 14;25(10):5331. doi: 10.3390/ijms25105331 (PMC11120685; doi:10.3390/ijms25105331)
Supplement: Supplementary file 1 [file ijms-25-05331-s001.zip › ijms-2949963-supplementary.pdf]

Supplementary Data

Machine Learning Approach on Metabolomic Data Predict T2DM Incidence

Andreas Leiherer [1,2,3*], Axel Muendlein [1,2], Silvia Mink [2,3], Arthur Mader [1,5], Christoph H Saely [1,2,5], Andreas Festa [1], Peter Fraunberger [3,2], And Heinz Drexel [1,2,4,6]

1 Vorarlberg Institute for Vascular Investigation and Treatment (VIVIT), Feldkirch, Austria
2 Private University of the Principality of Liechtenstein, Triesen, Liechtenstein
3 Central Medical Laboratories, Feldkirch, Austria
4 Vorarlberger Landeskrankenhausbetriebsgesellschaft, Academic Teaching Hospital Feldkirch, Feldkirch, Austria
5 Department of Internal Medicine III, Academic Teaching Hospital Feldkirch, Feldkirch, Austria
6 Drexel University College of Medicine, Philadelphia, PA, USA
* Correspondence: : E-mail: vivit@lkhf.at; Phone: +43 (0)5522 303 6902

Supplementary Tables

Supplementary Table S1. Comparison of most important variables regarding patients' outcome.

	**total**	**No T2DM incidence**	**T2DM incidence**	**p-value**
	n=279	n=247	n=32	
Hexoses	4450 [3971-4985]	4411 [3898-4871]	5194[4455-5451]	<0.001
Glycine	200 [172-241]	202 [177-243]	175 [160-209]	0.002
Isoleucine	67 [57-79]	66 [57-79]	76 [66-86]	0.002
CDCA	0.21 [0.07-0.61]	0.18 [0.07-0.59]	0.48 [0.15-1.32]	0.004
DCA	0.41 [0.15-1.06]	0.36 [0.14-1.50]	0.88 [0.24-1.76]	0.021
Kynurenine	4.13 [3.46-4.84]	4.04 [3.41-4.75]	4.55 [4.15-5.33]	0.006
UDCA	0.06 [0.03-0.12]	0.06 [0.03-0.11]	0.08 [0.05-0.20]	0.010
GUDCA	0.07 [0.03-0.14]	0.06 [0.03-0.12]	0.11 [0.04-0.22]	0.044
Tyrosine	68 [56-81]	67 [55-79]	75 [67-87]	0.003
N-C 18:1 Cer	7.0×10^{-3} [4.8×10^{-3}-9.9×10^{-3}]	7.1×10^{-3} [4.9×10^{-3}-10.2×10^{-3}]	6.4×10^{-3} [3.8×10^{-3}-7.8×10^{-3}]	0.072
Valine	190 [166-224]	188 [165-223]	203 [181-240]	0.030
LCA	0.02 [0.01-0.04]	0.02 [0.01-0.04]	0.03 [0.02-0.05]	0.050
CA	0.17 [0.05-0.69]	0.15 [0.05-0.55]	0.56 [0.09-0.84]	0.044
PE aa C:38:1	0.17 [0.14-0.20]	0.17 [0.14-0.20]	0.15 [012-0.17]	0.013
N-C 14:0 Cer	7.9×10^{-3} [5.6×10^{-3}-11.4×10^{-3}]	8.2×10^{-3} [5.7×10^{-3}-11.8×10^{-3}]	5.9×10^{-3} [5.1×10^{-3}-9.0×10^{-3}]	0.012
αKGA	21.8 [8.0-32.6]	21.4 [7.9-31.7]	24.8 [10.7-39.2]	0.191
PC ae C:38:5	11.8 [8.2-15.7]	11.7 [8.1-15.2]	12.6 [9.0-17.6]	0.208
N-C 15:0 Cer (H2)	0.8×10^{-3} [0.2×10^{-3}-1.4×10^{-3}]	0.8×10^{-3} [0.4×10^{-3}-1.4×10^{-3}]	0.6×10^{-3} [0-1.4×10^{-3}]	0.206
Lactic acid	925 [657-1.289]	930 [650-1268]	891 [678-1380]	0.553
PE ae C:40:6	1.03 [0.83-1.41]	1.05 [0.85-1.41]	0.97 [0.76-1.34]	0.130

High important variables (all continuous not normally distributed data) are given as median and interquartile range [IQR]. Differences between patients who have developed T2DM during the four-year follow-up (positive outcomes) and patients who did not develop T2DM during follow-up (negative outcomes) were tested with the Jonckheere-Terpstra test.

Supplementary Table S2. Correlation and collinearity analysis of important variables.

	r	p-value	VIF
Hexoses	0.254	<0.001	1.3
Glycine	-0.184	0.002	1.2
Isoleucine	0.185	0.002	4.3
CDCA	0.175	0.004	1.9
DCA	0.140	0.020	1.7
Kynurenine	0.165	0.006	1.5
UDCA	0.155	0.010	1.5
GUDCA	0.122	0.044	1.5
Tyrosine	0.177	0.003	1.8
N-C 18:1 Cer	-0.108	0.072	22105.2
Valine	0.130	0.030	4.7
LCA	0.125	0.050	1.3
CA	0.121	0.044	2.0
PE aa C:38:1	-0.149	0.013	29991.2
N-C 14:0 Cer	-0.151	0.012	26343.1
αKGA	0.079	0.191	1.8
PC ae C:38:5	0.076	0.209	285.0
N-C 15:0 Cer (H2)	-0.082	0.206	414.0
Lactic acid	0.036	0.554	1.3
PE ae C:40:6	-0.091	0.130	4428.5

The correlation (r) of high important variables with the outcome is given as nonparametric Spearman rank correlation (r) with the corresponding p-values. The multicollinearity between the features is given as Variance Inflation Factor (VIF).

Supplementary Table S3. Correlation of Important features with outcome.

	coefficient
(Intercept)	-5.108
Hexoses	4.147
Lactic acid	3.452
Glycine	-3.041
C14:2	1.441
Kynurenine	1.420
C16	-1.225
Isoleucine	0.734
GUDCA	0.596
Tyrosine	0.332
GLCAS	0.028
CDCA	0.012

The coefficients of the Lasso regression represent the relationship between each predictor variable (n=11) and the response variable (diabetes developed vs.- no diabetes developed), adjusted for the regularization penalty that Lasso applies. A positive coefficient suggests that as the predictor increases, the response variable also increases, assuming all other predictors are held constant. A negative coefficient indicates that as the predictor increases, the response variable decreases. Variables who were not used (n=66, named zero Coefficients) have been excluded from the model due to shrinking of their coefficient to exactly zero by Lasso regression.

Supplementary Figures

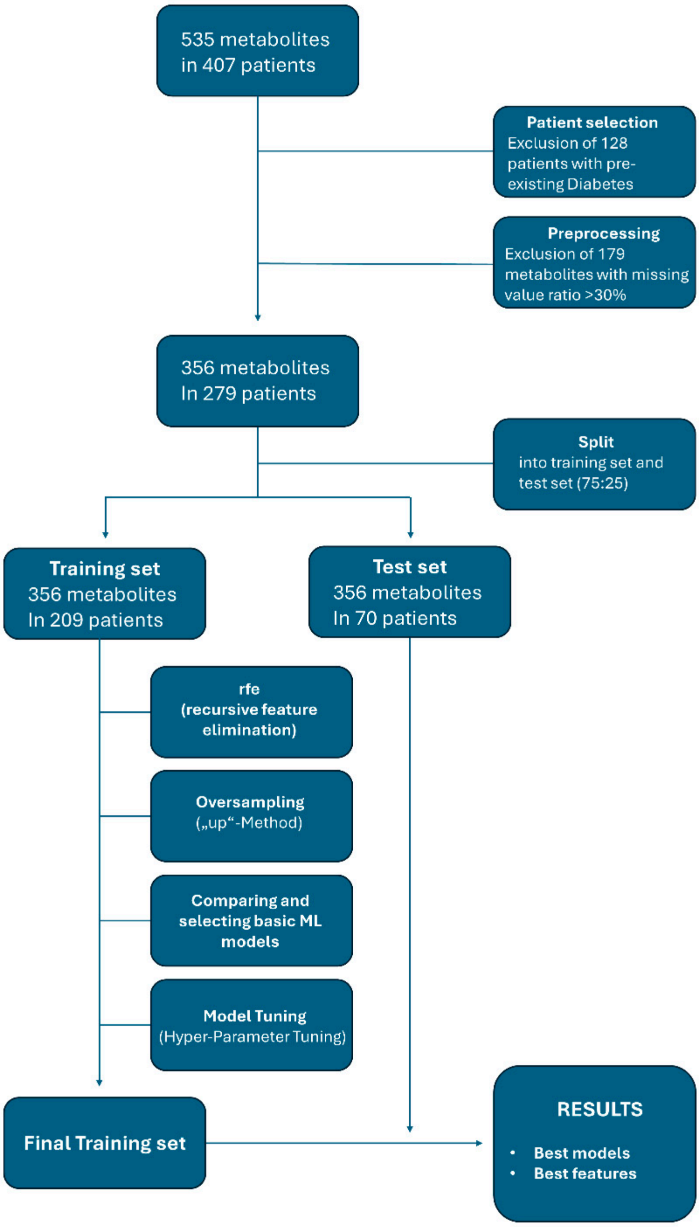

Supplementary figure S1 Analysis tree

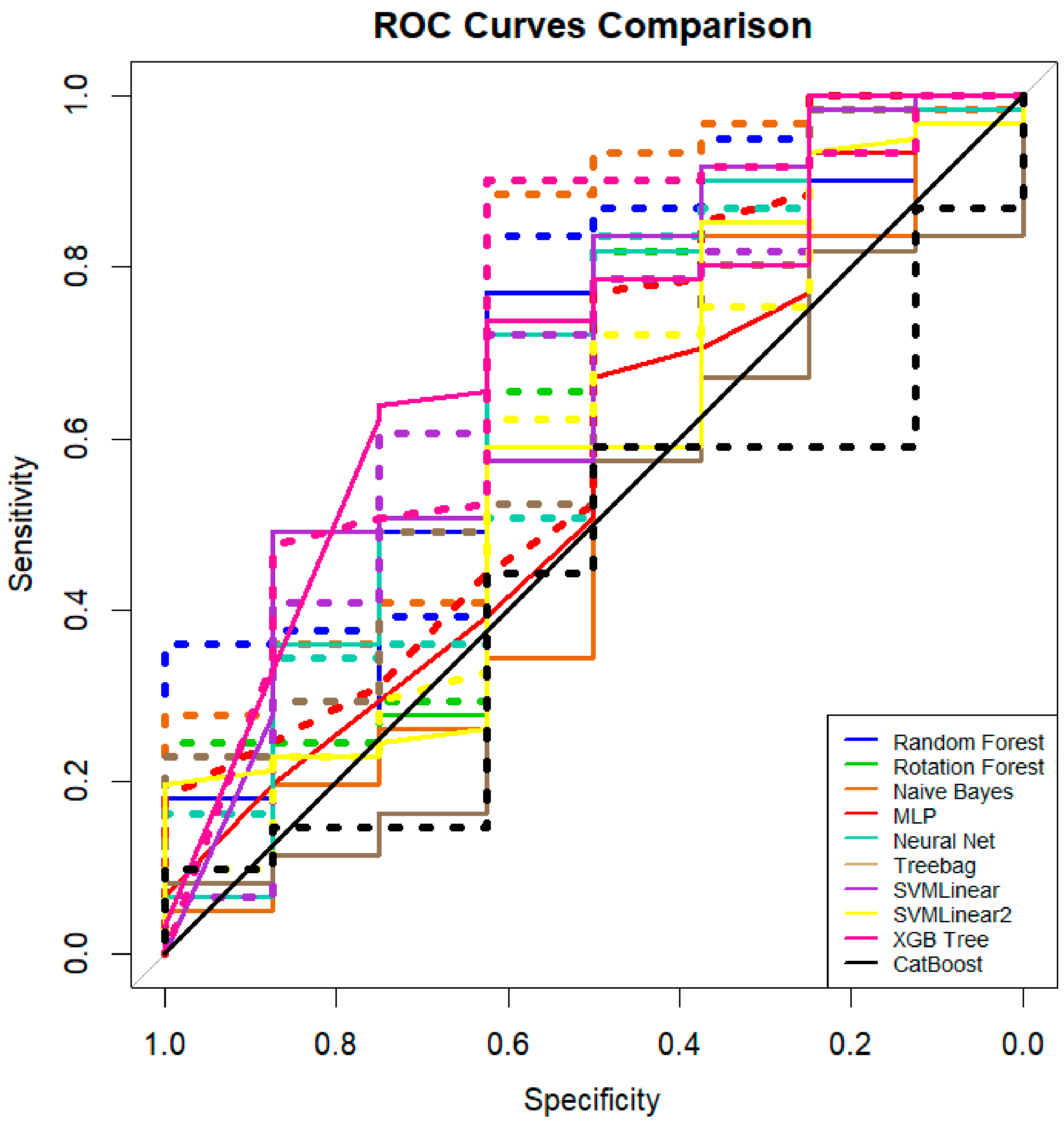

Supplementary figure S2: Receiver operating characteristic curves of different models. Solid lines represent models built with the full set of features (n=362), doted lines represent models with the selected set (n=77). The model set includes Random Forest, Rotation Forest with complexity parameter tuning (Rotation Forest) Naive Bayes, Multi-Layer Perceptron (MLP), feedforward Neural Network with Principal Component Analysis (Neural Net), Bootstrap Bagging of decision trees (Treebag), two variations of Support Vector Machines with linear kernels (SVMLinear and SVMLinear2), Extreme Gradient Boosting for tree-based models (XGB Tree), and an open-source Gradient Boosting on decision trees (CatBoost).

Supplementary Methods

R code for ML

```
### ML with Caret ###
# Install and load packages
# Using the set.seed() function is recommended
# Make sure to load basic packages ("ggplot2", "pROC",  "tidyr") in particular the caret package
library(caret)

# Import dataset, make sure it is a dataframe
# Missing Values threshold (select your threshold, e.g. 30%)
threshold <- 0.31
Imetn <- Imet[, colSums(is.na(Imet)) / nrow(Imet) < threshold]

# Split dataset in training and test
# Create the training and test datasets (e.g.75/25)
trainRowNumbers <- createDataPartition(Imetn$Kat, p=0.75, list=FALSE)
trainRowNumbers <- as.vector(trainRowNumbers)
trainData <- Imetn[trainRowNumbers,]
testData <- Imetn[-trainRowNumbers,]

# define X and Y for later use (given you have 363 variables and the first (“Kat”) is the outcome)
x = trainData[,2:363]
y = trainData$Kat

## Imputation of missing values
preProcess_missingdata_model <- preProcess(trainData, method='knnImpute')
preProcess_missingdata_model
# Use the imputation model to predict the values of missing data points
library(RANN)
trainData2 <- predict(preProcess_missingdata_model, newdata = trainData)

## One-Hot Encoding
dummies_model <- dummyVars(Kat ~ ., data=trainData2)
trainData2_mat <- predict(dummies_model, newdata = trainData2)
trainData3 <- data.frame(trainData2_mat)

## Preprocessing
preProcess_range_model <- preProcess(trainData3, method='range')
trainData4 <- predict(preProcess_range_model, newdata = trainData3)

# Append the Y variable
trainData4$Kat <- y

## recursive feature elimination (rfe)
# Select subset for rfe (e.g. all combinations allowed)
subset_max <- c(1:362)
# define the model (e.g. random forest with repeatedcv)
ctrl <- rfeControl(
                functions = rfFuncs,
                 method = "repeatedcv",
                 repeats = 5,
                 verbose = FALSE)
lmProfile <- rfe(x=trainData4[, 1:362], y=trainData4$Kat,
               sizes = subset_max,
               rfeControl = ctrl,
                )
```

```
# See output
print(lmProfile)
ggplot(data = lmProfile, metric = "Accuracy") + theme_bw()

# Prepare test data set the same way
testData2 <- predict(preProcess_missingdata_model, testData)
testData3 <- predict(dummies_model, testData2)
testData3 <- data.frame(testData3)
testData4 <- predict(preProcess_range_model, testData3)

# Creating a new data set with only variables selected by rfe
selected_features <- predictors(lmProfile)
trainData4_selected <- trainData4[, selected_features]
testData4_selected <- testData4[, selected_features]
trainData4_selected$Kat <- y

## Generating a ML  model
# make sure your categorical variable is a factor
# using the set.seed() function is recommended
# example: MLP
fitControl <- trainControl(
  method = 'cv',
  number = 5,
  savePredictions = 'final',
  classProbs = T,
  summaryFunction=twoClassSummary  # results summary function
)

fitControl_up <- trainControl(
  method = 'cv',
  number = 5,
  savePredictions = 'final',
  classProbs = T,
  summaryFunction=twoClassSummary,
  sampling = "up"                # Oversampling method: "up"
)

# define your grid (e.g size = 1:40
param_grid <- expand.grid(size = 1:40)

# generate models (data set either full data set (traindData4) or rfe-selected (traindData4_selected))
model_mlp = train(Kat ~ ., data=trainData4, method='mlp', trControl = fitControl)
# tune and modify models e.g.:
#model_mlp_up = train(Kat ~ ., data=trainData4, method='mlp', trControl = fitControl_up)
#model_mlp_grip = train(Kat ~ ., data=trainData4, method='mlp', trControl = fitControl,  #tuneGrid=param_grid)
#model_mlp_grip_up = train(Kat ~ ., data=trainData4, method='mlp', trControl = fitControl_up, #tuneGrid=param_grid)
predicted_mlp <- predict(model_mlp, testData4)
predicted_prob_mlp <- predict(model_mlp, testData4, type = "prob")
confusionMatrix(reference = testData$Kat, data = predicted_mlp, mode='everything')
roc_mlp <- roc(response = testData$Kat, predictor = predicted_prob_mlp[, "X1"],levels = c("X1", "X0"))
prob_mlp_auc_value <- auc(roc_mlp)
print(prob_mlp_auc_value)
varimp_mlp <- varImp(model_mlp)
plot(varimp_mlp, top = 20, main="Variable Importance with mlp")
```

R code for SHAP

```
### SHAP ###
# Load the SHAP package
library(shapviz)
```

```
library(shapviz)
library(kernelshap)
xvars <- colnames(trainData4_selected[,1:77])
xvars_full <- colnames(trainData4[,1:362])
s <- kernelshap(model_s_svml2, trainData4_selected, predict, bg_X = trainData4_selected, feature_names = xvars)
s_full <- kernelshap(model_svml2_up, trainData4, predict, bg_X = trainData4, feature_names = xvars)
sv <- shapviz(s)
sv_importance(sv, kind = "beeswarm")
sv_importance(sv)
```

R code for Lasso Regression

```
### Lasso-Regression (alpha=1) ###
# Define a subset of relevant variables (e.g. n=77) in your data set (RV1, RV2, RV3, … RV77)
important_vars <- c("RV1", "RV2", "RV3", "RV4", "RV5", "RV6",
            "RV7", "RV8", "RV9", "RV10", "RV11", "RV12", "RV13",
            "RV14", "RV15", "RV16”, "RV17", "RV18", "RV19”, "RV20",
                    RV21", "RV22", "RV23", "RV24", "RV25", "RV26",
            "RV27", "RV28", "RV29", "RV30", "RV31", "RV32", "RV33",
            "RV34", "RV35", "RV36”, "RV37", "RV38", "RV39”, "RV40
                   RV41", "RV42", "RV43", "RV44", "RV45", "RV46",
            "RV47", "RV48", "RV49", "RV50", "RV51", "RV52", "RV53",
            "RV54", "RV55", "RV56”, "RV57", "RV58", "RV59”, "RV60",
                    RV61", "RV62", "RV63", "RV64", "RV65", "RV66",
            "RV67", "RV68", "RV69", "RV70", "RV71", "RV72", "RV73",
            "RV74", "RV75", "RV76”, "RV77")

# Check scale of data
# Define data set and outcome variable (Kat)
y <- as.numeric(as.factor(trainData$Kat)) - 1
x <- as.matrix(trainData)

# Build and check models
fit <- glmnet(x, y, family = "binomial", alpha = 1)  # alpha=1 für Lasso
cv_fit <- cv.glmnet(x, y, family = "binomial", alpha = 1, nfolds = 10)
plot(cv_fit)

# Choosing Lamda
cv_fit$lambda
best_lambda <- cv_fit$lambda.min

# Check best_lambda (modify if necessary)
best_lambda

# Display Coefficinets for final model with seleted Lambda
final_model <- glmnet(x, y, family = "binomial", alpha = 1, lambda = best_lambda)
coefficients <- coef(final_model, s = best_lambda)
print(coefficients)
```

R code for VIF calculation

```
### VIF ###
# Load necessary library
library(car)

# Check class of dependent variable
View(Imet)
class(Imet$Kat)
Imet$Kat <- as.numeric(Imet$Kat)
```

```
class(Imet$Kat)

# Fit a linear model with “important variables” (eg IV1, IV2, IV3…)
model <- lm(Kat ~ IV1 + IV2 + IV3 + IV4 + IV5 + IV6 + IV7 + IV8 + IV9 + IV10 + IV11 + IV12 + IV13 + IV14 + IV15 + IV16 + IV17 +
IV18 + IV19 + IV20, data = Imet)

# Calculate VIF
vif_vals <- vif(model)
print(vif_vals)
```
